# Impact of sampling and data collection methods on maternity survey response: a randomised controlled trial of paper and push-to-web surveys and a concurrent social media survey

**DOI:** 10.1186/s12874-023-01833-8

**Published:** 2023-01-12

**Authors:** Siân Harrison, Fiona Alderdice, Maria A. Quigley

**Affiliations:** NIHR Policy Research Unit in Maternal and Neonatal Health and Care, National Perinatal Epidemiology Unit, Nuffield Department of Population Health, University of Oxford, Old Road Campus Headington, Oxford, OX3 7LF UK

**Keywords:** Survey, Questionnaire, Sampling, Response rate, Representativeness, Non-response bias, Selection bias, Weighting, Pregnancy, Maternity, Randomised controlled trial (RCT)

## Abstract

**Background:**

Novel survey methods are needed to tackle declining response rates. The 2020 National Maternity Survey included a randomised controlled trial (RCT) and social media survey to compare different combinations of sampling and data collection methods with respect to: response rate, respondent representativeness, prevalence estimates of maternity indicators and cost.

**Methods:**

A two-armed parallel RCT and concurrent social media survey were conducted. Women in the RCT were sampled from ONS birth registrations and randomised to either a paper or push-to-web survey. Women in the social media survey self-selected through online adverts. The primary outcome was response rate in the paper and push-to-web surveys. In all surveys, respondent representativeness was assessed by comparing distributions of sociodemographic characteristics in respondents with those of the target population. External validity of prevalence estimates of maternity indicators was assessed by comparing weighted survey estimates with estimates from national routine data. Cost was also compared across surveys.

**Results:**

The response rate was higher in the paper survey (*n* = 2,446) compared to the push-to-web survey (*n* = 2,165)(30.6% versus 27.1%, difference = 3.5%, 95%CI = 2.1–4.9, *p* < 0.0001). Compared to the target population, respondents in all surveys were less likely to be aged < 25 years, of Black or Minority ethnicity, born outside the UK, living in disadvantaged areas, living without a partner and multiparous. Women in the social media survey (*n* = 1,316) were less representative of the target population compared to women in the paper and push-to-web surveys. For some maternity indicators, weighted survey estimates were close to estimates from routine data, for other indicators there were discrepancies; no survey demonstrated consistently higher external validity than the other two surveys. Compared to the paper survey, the cost saving per respondent was £5.45 for the push-to-web survey and £22.42 for the social media survey.

**Conclusions:**

Push-to-web surveys may cost less than paper surveys but do not necessarily result in higher response rates. Social media surveys cost significantly less than paper and push-to-web surveys, but sample size may be limited by eligibility criteria and recruitment window and respondents may be less representative of the target population. However, reduced representativeness does not necessarily introduce more bias in weighted survey estimates.

## Background

Surveys are an important method for collecting health-related data, particularly data which are not routinely available from other sources. Population-based surveys often use random sampling to select respondents and employ a variety of data collection methods, such as structured interviews or questionnaires administered via post or online. Irrespective of the methodology, there has been a steady decline in response rates to surveys over recent decades [1, 2]. The declining trend is exemplified in the National Maternity Surveys (NMS) which use postal questionnaires to survey postnatal women in England, and in which the response rate has fallen from 67% in 1995 to 29% in 2020 [3].

Surveys with low response rates are less likely to be representative of their target population [4], which may introduce bias in the estimates based on the data collected [5]. It is important, therefore, to identify survey characteristics which optimise returns and increase the likelihood of obtaining representative samples. One characteristic that has been investigated in surveys with defined sampling frames is survey mode, and a recent meta-analysis of 114 experimental comparisons concluded that online surveys yield lower response rates than other modes, such as postal or telephone surveys [6]. Despite the lower rates of response, the growth of online surveys is accelerating, either as an alternative or as a complement to postal surveys [7]. When sampling frames preclude online invitation, due to unavailability of email data, a ‘push-to-web’ methodology can be used, whereby the survey invitation is sent by post with a link to the online survey, and the offer of participation via post is withheld until a later contact [8]. Push-to-web methods have been shown to increase response rates and reduce costs [9, 10], but evidence and support for this methodology remains mixed [8, 11, 12].

Since the early 2000s there has been a rapid increase in online surveys recruiting through social media and often with no sampling frame [4]. Such surveys have the potential to recruit large numbers of respondents at a fraction of the cost compared with selecting participants from sampling frames and administering postal, online or push-to-web surveys. There are, however, several potential biases arising from the recruitment of survey participants through social media surveys and with no defined sampling frame. These include under-coverage, with participation being limited to those who have access to the internet and who visit the relevant webpage or social media platform, and self-selection, with participants self-identifying and choosing to take part [13]. Furthermore, without a sampling frame, inferences from the data are limited because there is seldom a description of the representativeness of the sample.

Bias due to non-response can be mitigated to some extent by applying correction techniques such as survey weighting, whereby the sample is weighted on certain characteristics in order to more accurately reflect the target population [14]. Survey weights are usually derived using data on non-respondents if available (non-response weights), or on the wider population from which the sample was drawn (post-stratification weights) [15]. However, survey weights tend not to be used in online surveys which recruit through social media and it is not known whether these correction techniques can reduce bias due to self-selection in samples with no sampling frame.

The 2020 NMS included an RCT (with standard paper and push-to-web survey arms) and a concurrent social media survey. The primary objective (of the RCT only) was to compare response rates between the paper survey and the push-to-web survey. The secondary objectives were to compare the paper survey, push-to-web survey and social media survey with respect to: respondent representativeness; external validity of prevalence estimates of key maternity indicators (compared with the same indicators in national routine data); and financial costs.

## Methods

This manuscript is written in line with the CONSORT guidelines (see Related File 1 for completed checklist).

### Design and participants

The 2020 NMS was conducted by the Policy Research Unit in Maternal and Neonatal Health and Care at the National Perinatal Epidemiology Unit (NPEU) and full details about the study methods are published separately [3]. The 2020 NMS included a two-armed parallel RCT and a social media survey which was conducted alongside the RCT. The study characteristics of the 2020 NMS are described in Table 1.

**Table 1:** Study characteristics of the 2020 NMS

	**Paper survey (RCT arm 1)**	**Push-to-web survey (RCT arm 2)**	**Social media survey**
Design			
Survey design	Cross-sectional population-based postal survey	Cross-sectional population-based postal survey	Cross-sectional online survey
Sampling method	Random sample	Random sample	Self-selected sample
Allocation ratio	Simple 1:1 ratio	Simple 1:1 ratio	NA
Randomisation and blinding			
Sequence generation	Stratified block randomisation using Microsoft Access VBA	Stratified block randomisation using Microsoft Access VBA	NA
Allocation concealment mechanism	None	None	NA
Implementation	By ONS	By ONS	NA
Blinding	None	None	NA
Participants			
Sampling frame	All births registered in England between 11^th^-24^th^ May 2020	All births registered in England between 11^th^-24^th^ May 2020	None
Eligibility criteria	Gave birth in England during May 2020 Aged ≥ 16 years Living in England when birth registered	Gave birth in England during May 2020 Aged ≥ 16 years Living in England when birth registered	Gave birth in UK between March and August 2020 Aged ≥ 16 years Living in UK when birth registered
Identification	By ONS (from birth registration records)	By ONS (from birth registration records)	Self-identified (through online adverts)
Recruitment period	November 2020 – March 2021	November 2020 – March 2021	November 2020 – February 2021
Target sample size	8,025	8,025	No target sample size
Intervention			
Type of survey invitation	Postal invitation with paper questionnaire (standard method) ^*^	Postal invitation with link to online survey (push-to-web method) ^*^	Advert (not randomised)
Outcomes			
Primary	Response rate	Response rate	NA ^**^
Secondary	Respondent representativeness External validity of prevalence estimates Cost	Respondent representativeness External validity of prevalence estimates Cost	Respondent representativeness External validity of prevalence estimates Cost

^*^ In the paper survey, women received a paper questionnaire with the option to take part online; in the push-to-web survey, women received an invitation to take part online but a paper questionnaire was included with the final reminder

^**^Response rate in the social media survey could not be calculated due to the denominator being unknown although the number of responses was presented

### RCT: Paper survey and push-to-web survey

The paper and push-to-web surveys were both cross-sectional population-based postal surveys carried out in England. The Office for National Statistics (ONS) identified 16,050 women from birth registration records in England and randomised these women to either the paper survey or the push-to-web survey. Stratified block randomisation of women based on region of residence in England was applied in a ratio of 1:1 and sequence generation was determined by ONS using Microsoft Access Visual Basic for Applications (VBA) code. Blinding and allocation concealment were not possible. The paper and push-to-web surveys each included a random sample of 8,025 postpartum women, which was representative of the target population: all women aged 16 years or older who had given birth in England during a two-week period in May 2020, and who were living in England at the time the birth was registered. Both the paper and push-to-web surveys involved contacting women by post with up to three mailings: an initial invitation (in November 2020), a reminder after 2–3 weeks (in December 2020), and a final reminder after a further 4–5 weeks (in January 2021).

In the paper survey, women received an invitation letter, participant information sheet, multi-language information sheet, a paper questionnaire and a reply-paid envelope with each mailing. The invitation letter in the paper survey also included details of how women could take part online using a web address or by scanning a QR code, if this mode of response was preferred. In the push-to-web survey, women received an invitation letter, participant information sheet and multi-language information sheet but the inclusion of a paper questionnaire and reply-paid envelope were withheld until the third (and final) mailing. Again, details of how to access the online survey were provided in the invitation letter, along with details of how to obtain a paper questionnaire, if this mode of response was preferred. Responses in the paper survey and the push-to-web survey were accepted until the end of March 2021 and all respondents were offered the option to receive a £5 shopping voucher by providing their email address, either on paper or online.

### Social media survey

The social media survey was a cross-sectional online survey carried out in the UK and included a self-selecting sample of women recruited by advertising on social media platforms: Facebook, Instagram, Twitter and Pinterest. There was no sampling frame; the survey was open to all women aged 16 years or older, who had given birth in the UK between March and August 2020, an estimated 340,780 women (292,598 in England), and who were living in the UK at the time of the survey [16]. Women could access the social media survey by clicking on a link to a web address within the survey advert. Women were first taken to an online participant information sheet and then to a series of self-screening questions to confirm their eligibility to participate. Eligible women were invited to complete the questionnaire online. The eligibility criteria for the social media survey were wider than for the paper or push-to-web surveys to enable this novel method of recruiting to the national maternity surveys to be explored more fully. In addition, wider criteria were needed to increase the likelihood that women viewing and responding to the adverts on social media platforms would be eligible to take part. The social media survey was open from the end of November 2020 until the end of February 2021, approximately three months in total. Respondents in the social media survey were offered the option to be entered into a prize draw for a chance to win one of five £100 shopping vouchers.

### Questionnaire data

The questionnaire content was identical in the paper, push-to-web and social media surveys. Unified mode construction principles were followed in designing the paper and online questionnaires in order to produce parallel tools with unified branding [17]. Women self-reported sociodemographic characteristics, physical and mental health, and maternity experiences during pregnancy, labour and birth, and the postnatal period. Some additional anonymised sociodemographic information was provided by ONS for women in the paper and push-to-web surveys, for example, region of residence and level of area-based deprivation measured by the Index of Multiple Deprivation (IMD). Women in the social media survey were asked to provide their postcode during the eligibility screen in order to derive their country and region of residence and their IMD.

### Outcomes

The primary outcome (for the RCT only) was survey response rate, compared across the paper and push-to-web surveys. The response rates were calculated by dividing the total number of responses (excluding refusals, duplicate and unusable returns) by the total number of women sampled (excluding packs confirmed as undelivered). Separate postal and online response rates were also calculated for the paper and push-to-web surveys.

The secondary outcomes were: respondent sociodemographic characteristics (age, ethnicity, country of birth, IMD, region of residence, cohabiting status (e.g. living with partner), education, parity and multiplicity); prevalence of key maternity indicators (homebirth, preterm birth, low birthweight, caesarean section); and financial cost (total cost and cost per respondent). These secondary outcomes were compared across the paper, push-to-web and social media surveys (only women living in England in the social media survey). All outcomes were assessed when data collection closed, which was the end of February in the social media survey and the end of March in the paper and push-to-web surveys.

### Statistical analysis

All analyses were conducted in Stata version 17.6 [18]. The overall, postal and online response rates in the paper and push-to-web surveys were calculated and the differences were estimated with 95% confidence intervals (CI) and compared using Chi-square tests. A nominal response rate was also calculated for the social media survey using the estimated number of births in England during March to August 2020 (*N* = 292,598, 50% of total births in England during 2020). The overall numbers of usable responses were compared across the paper, push-to-web and social media surveys.

The distributions of respondent sociodemographic characteristics (listed under outcomes) for the women who responded in the paper, push-to-web and social media surveys were described and compared using Chi-square tests. The representativeness of the respondents in each survey was assessed by comparing the distributions of sociodemographic characteristics to those for all women who gave birth in England during 2020 (with the exception of education, which is unavailable at population-level).

Due to sociodemographic differences between the respondents and non-respondents in the paper and push-to-web surveys, and the potential impact of these differences on prevalence estimates based on survey data, non-response survey weights were derived for the paper and push-to-web surveys using data on non-respondents provided by ONS [3]. The variables used to create the non-response weights were age, registration status (registered in married, joint or single names), country of birth, IMD, region of residence and parity [3]. Post-stratification survey weights were derived for the social media survey using data on the population of all women giving birth in the UK in 2019, which were the most recent national routine data available when the survey weights were constructed. The variables used to create the post-stratification weights were age, country of birth, IMD, country of residence and parity [3]. The derived non-response and post-stratification survey weights were then applied to the data in the paper and push-to web surveys and the social media survey, respectively. The weighted prevalence estimates with 95% CIs of key maternity indicators were estimated using the survey commands in Stata. The external validity was assessed by comparing the weighted estimates and 95% CI across the surveys and with population-based prevalence estimates available from national routine data. The key maternity indicators were proportions for: homebirth, low birthweight, preterm birth, and caesarean section.

Finally, the total financial costs were calculated and compared (in 2020/21 UK£) across the paper, push-to-web and social media surveys. The main costs incurred in the paper and push-to-web surveys were administrative costs for ONS to draw the sample from birth registration records, supply and printing of paper questionnaires and study documents, mail-out and return postage, data capture and supply, design and management of the online survey, and individual incentives for respondents. The main costs incurred in the social media survey were for the design and management of the online survey, advertising the survey on social media platforms, and the prize draw incentives. No financial discounts were received when purchasing the various resource components required to administer the surveys and so costs likely reflect market prices and could be generalisable to similar types of surveys. The cost per respondent was calculated for the paper, push-to-web and social media surveys by dividing the total cost for each survey by the number of usable responses in each survey (for women living in England only in the social media survey).

### Sample size

The combined sample size for the paper and push-to-web surveys was calculated to ensure that: 1) prevalence of key survey outcomes (e.g. health and care outcomes) could be estimated with adequate precision; and 2) a small difference in response rates could be detected between the paper and push-to-web surveys. Assuming women were randomised equally to the two survey arms and a baseline response rate of 29% (based on previous NMS, [19]), we calculated that a sample size of 16,050 women (8,025 women in each arm) would be sufficient to provide approximately 90% power to estimate a range of effects and to compare key outcomes in different groups of women (reported separately [3]) and > 95% power to detect a between-group difference in response rate of at least 3% (reported here).

## Results

### Response rate

**Fig. 1:**
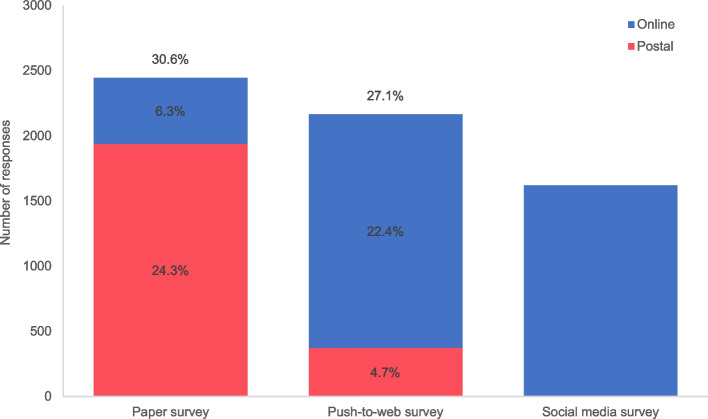
Number and proportion of overall, postal and online responses across the three surveys

In total, 2,446 out of 7,992 (30.6%) women responded in the paper survey and 2,165 out of 7,980 (27.1%) women responded in the push-to-web survey (Fig. 1). The response rate in the paper survey was significantly higher than in the push-to-web survey (+ 3.5%, 95%CI: 2.1 to 4.9, *p* < 0.0001). The majority of women in the paper survey opted to complete and return the paper questionnaire (*n* = 1,940, 24.3%) rather than to take part online (*n* = 506, 6.3%). Conversely, the majority of women in the push-to web survey opted to complete and return the online questionnaire (*n* = 1,790, 22.4%) rather than to take part on paper after receiving the final reminder (*n* = 375, 4.7%). The CONSORT flow diagram for the RCT is shown in Related File 2.

In total, 1,316 women (who were living in England) responded in the social media survey (1,622 including those women living in Northern Ireland, Scotland and Wales). The total number of births in England during the data collection period was 292,598, and so the nominal response rate in the social media survey was 0.4% (1,316 out of 292,598). In the following analyses, the social media survey only includes those women who were living in England at the time of survey participation.

### Representativeness of respondents

**Fig. 2:**
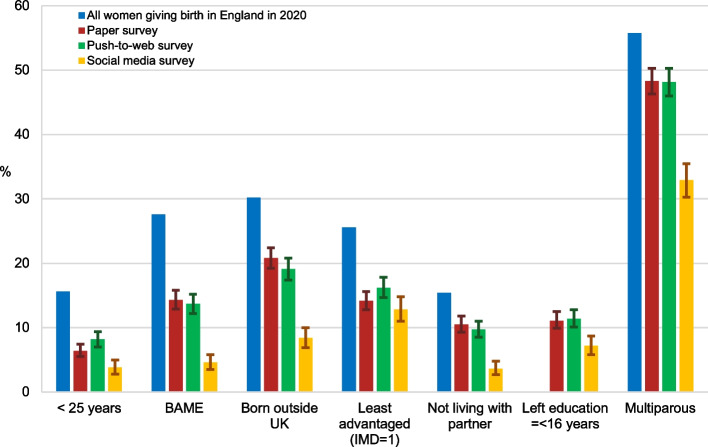
Proportion of women (with 95% CI) with different sociodemographic characteristics across the three surveys

The survey respondents, regardless of the specific survey methods used, differed to the target population on key sociodemographic characteristics, with more marked differences between the women recruited through social media and the women in the target population (Table 2). In particular, compared to all women who gave birth in England in 2020, the survey respondents were less likely to be aged < 25 years old, of Black or Minority ethnicity, born outside of the UK, living in less advantaged areas in England, living without or separately from a partner, and multiparous; and the 95% CIs for the prevalence of these characteristics did not include the target population prevalence (Fig. 2). The women who took part in the paper survey were similar to those who took part in the push-to-web survey, with the exception that the women in the push-to-web survey were more likely to be living in less advantaged areas (16.2% in 1^st^ IMD quintile in push-to-web survey versus 14.2% in 1^st^ IMD quintile in paper survey (*p* = 0.045)). However, the women who took part in the social media survey were significantly different to the women who took part in the paper and push-to-web surveys on all sociodemographic variables (*p* < 0.05) with the exception of multiplicity (2.4% multiple birth rate in social media survey versus 1.6% in paper survey (*p* = 0.214) and 1.4% in push-to-web survey (*p* = 0.238)).

**Table 2:** Sociodemographic characteristics of the respondents across the three surveys and of all women giving birth in England in 2020

Population-level data for England^#^ *N* = 585,195		Paper survey *N* = 2,446		Push-to web survey *N* = 2,165		Social media survey *N* = 1,316^*^	
	**%**	**n**	**%**	**n**	**%**	**n**	**%**
**Age (years)**	*N* = 613,844^	*N* = 2,408		*N* = 2,154		*N* = 1,315	
16–19	2.6	11	0.5	14	0.6	3	0.2
20–24	13.0	143	5.9	162	7.5	47	3.6
25–29	26.8	539	22.4	436	20.2	327	24.9
30–34	33.5	912	37.9	866	40.2	583	44.3
35–39	19.3	634	26.3	545	25.3	286	21.7
40 +	4.8	169	7.0	131	6.1	69	5.2
**Ethnicity**^**†**^	*N* = 584,509	*N* = 2410		*N* = 2138		*N* = 1,313	
White	72.4	2,065	85.7	1,846	86.3	1,253	95.4
Asian	13.0	208	8.6	172	8.0	19	1.4
Black	5.2	69	2.8	57	2.6	6	0.5
Mixed / Other	9.5	68	2.8	63	2.9	35	2.7
**Country of birth**	*N* = 585,195	*N* = 2,403		*N* = 2,157		*N* = 1,316	
UK	69.8	1,904	79.2	1,746	80.9	1,206	91.6
Outside UK	30.2	499	20.8	411	19.1	110	8.4
**IMD**	*N* = 585,195	*N* = 2,446		*N* = 2,165		*N* = 1,287	
1^st^ (least advantaged)	25.6	347	14.2	351	16.2	165	12.8
2^nd^	22.4	463	18.9	413	19.1	211	16.4
3^rd^	19.5	487	19.9	470	21.7	314	24.4
4^th^	17.3	581	23.8	489	22.6	312	24.2
5^th^ (most advantaged)	15.3	568	23.2	442	20.4	285	22.1
**Living with partner**^**+**^	*N* = 585,195	*N* = 2,446		*N* = 2,165		*N* = 1,316	
Yes	84.6	2,189	89.5	1,955	90.3	1,268	96.4
No	15.4	257	10.5	210	9.7	48	3.6
**Age when leaving education**		*N* = 2,416		N = 2,147		N = 1,313	
16 years or younger	NA	269	11.1	245	11.4	94	7.2
17–18 years	NA	660	27.3	566	26.4	303	23.1
19 years or older	NA	1,487	61.5	1,336	62.2	916	69.8
**Region**	*N* = 585,195	*N* = 2,446		*N* = 2,165		*N* = 1,306	
North East	4.3	107	4.4	100	4.6	55	4.2
North West	13.0	256	10.5	258	11.9	133	10.2
Yorkshire & the Humber	9.6	221	9.0	195	9.0	118	9.0
East Midlands	8.0	200	8.2	164	7.6	90	6.9
West Midlands	10.9	255	10.4	218	10.1	158	12.1
East of England	11.0	298	12.2	243	11.2	158	12.1
London	19.1	398	16.3	370	17.1	147	11.3
South East	15.5	435	17.8	389	18.0	236	18.1
South West	8.6	276	11.3	228	10.5	211	16.2
**Parity**	*N* = 613,936^^^	*N* = 2,363		*N* = 2,144		*N* = 1,314	
Primiparous	44.2	1,222	51.7	1,111	51.8	882	67.1
Multiparous	55.8	1,141	48.3	1,033	48.2	432	32.9
**Multiplicity**	*N* = 613,936^^^	*N* = 2,426		*N* = 2,162		*N* = 1,314	
Single birth	98.6	2,388	98.4	2,131	98.6	1,283	97.6
Multiple birth	1.4	38	1.6	31	1.4	31	2.4

^*^In the social media survey, the majority (1,316 out of 1,622, 81.1%) of women were living in England at the time of survey participation and only these women were included in the analysis

^#^Distribution similar to ONS sample (respondents and non-respondents) for two week period in May 2020 – biggest percentage difference = 0.6%

^^^Data for England and Wales combined

^†^Ethnicity relates to baby for population-level data and to mother for survey data

^+^Based on registration status (yes = registered in married names or joint names, same address) for population-level data

### External validity of prevalence estimates

**Fig. 3:**
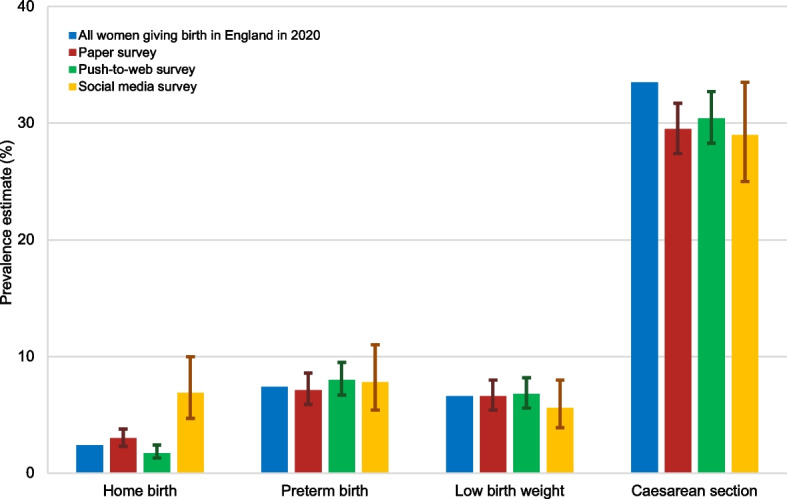
Weighted prevalence estimates (with 95% CI) of selected maternity indicators

Population-based estimates of selected maternity indicators based on national routine data are available from published reports and these estimates were used to assess the external validity of the prevalence estimates based on data from the three surveys (Table 3 and Fig. 3). For most of the indicators, the weighted survey estimates are close to the population-based estimates. For the proportion of babies who were born preterm and with low birth weight, the 95% CIs for the estimates from all surveys included the population-based estimate. For home birth, the population-based estimate was on the lower limit of the 95% CIs for the paper survey estimate and the upper limit of the 95% CIs for the push-to-web survey estimate, yet the 95% CIs for the estimate from the social media survey did not include the population-based estimate. For caesarean birth, the population-based estimate was on the upper limit of the 95% CIs for the social media survey estimate, yet the 95% CIs for the estimates from the paper and push-to-web surveys did not include the population-based estimate. Taken together, the results do not indicate that the weighted prevalence estimates from the paper, push-to-web or social media survey were consistently higher (than the other two surveys) in terms of the external validity. Furthermore, with the exception of home birth, the 95% CIs for the survey estimates of all maternity indicators overlap for the three surveys.

**Table 3:** Weighted prevalence estimates of selected maternity indicators from the three surveys compared to population-based prevalence estimates

	Population-based data for England % (data source, completeness %)		Paper survey weighted % (95%CI) *N* = 2,446		Push-to-web survey weighted % (95%CI) *N* = 2,165		Social media survey weighted % (95%CI) *N* = 1,316	
Home birth^†^	2.4	(ONS, 99.0^*^)	3.0	(2.3–3.8)	1.7	(1.3–2.4)	6.9	(4.7–10.0)
Preterm birth (< 37 weeks)	7.4	(ONS, 99.6^*^)	7.1	(5.9–8.6)	8.0	(6.7–9.5)	7.8	(5.5–11.0)
Low birth weight (< 2500 g)	6.6	(ONS, 97.3^*^)	6.6	(5.4–8.0)	6.8	(5.6–8.2)	5.6	(3.9–8.0)
Caesarean birth	33.5	(HES, 98.0^)	29.5	(27.4–31.7)	30.4	(28.3–32.7)	29.0	(25.0–33.5)

*ONS* Office for National Statistics, *HES* Hospital Episode Statistics

^*^ Completeness of data based on all births in England in 2020

^^^ Completeness of data based on all births in NHS hospitals in England in 2020

^†^ Data for England and Wales combined

### Financial cost

**Fig. 4:**
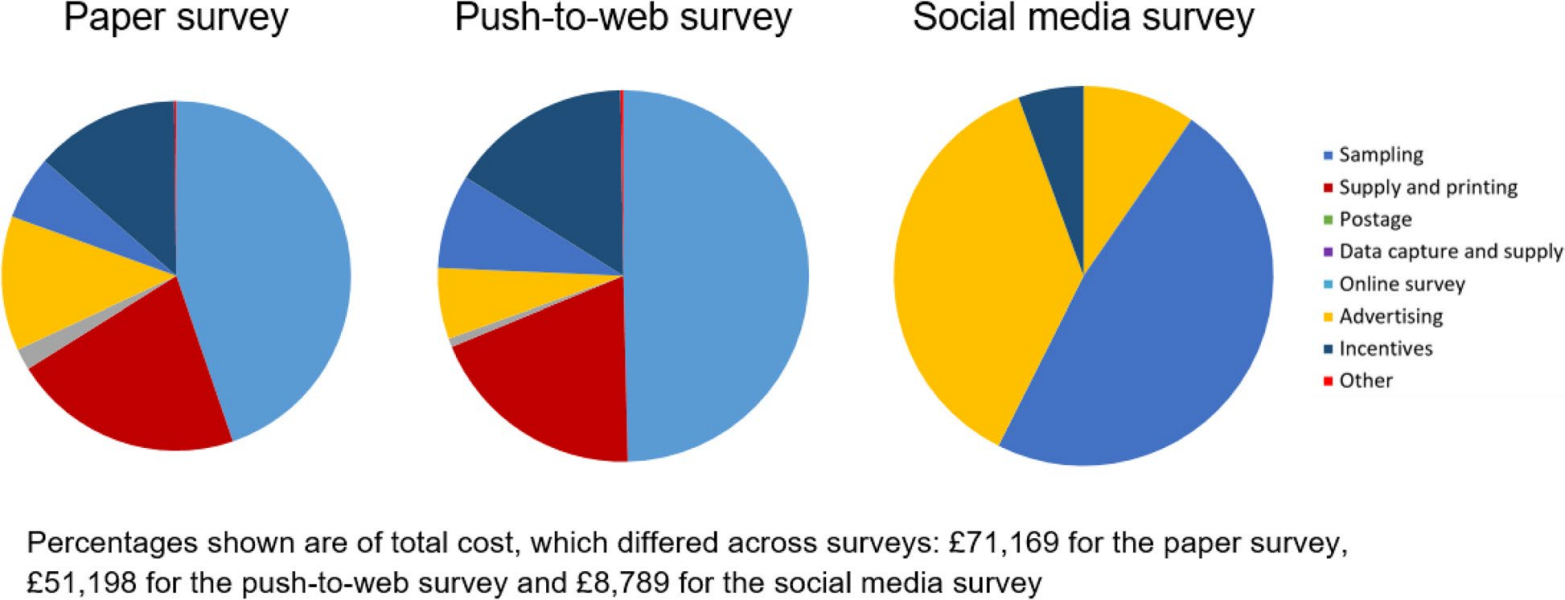
Breakdown of total costs across the surveys

The total costs were £71,169 for the paper survey compared to £51,198 for the push-to-web survey and £8,789 for the social media survey (Table 4). A breakdown of the total costs by key cost component for each of the surveys is shown in Fig. 4. The largest cost components of both the paper and push-to-web surveys were for ONS to draw the sample (45% and 50% of total cost in paper and push-to-web surveys, respectively), and for the supply and printing of paper study documents (21% and 19% of total cost in paper and push-to-web surveys, respectively); these costs were not applicable in the social media survey. All three surveys incurred the same absolute costs with regard to designing and managing the online component of the survey, yet for the social media survey, which had the lowest overall costs, this element made up the greater proportion of the total cost (48% of total cost in social media survey compared to 6–8% in paper and push-to-web surveys). The second largest cost component of the social media survey was advertising costs (37% of total cost), which were not applicable in the paper or push-to-web surveys. Data capture and supply costs and incentives for respondents were applicable across all surveys, albeit lower in absolute cost in the social media survey (e.g. the cost of incentives was £9,539 in the paper survey, £8,119 in the push-to-web survey and £490 in the social media survey) (Fig. 4).

**Table 4:** Financial cost of the three surveys

	**Paper survey *N* = 2,446**	**Push-to-web survey *N* = 2,165**	**Social media survey *N* = 1,316**
Total cost	£71,169	£51,198	£8,789
Total cost saving ^*^	NA	£19,971	£62,380
Cost per respondent	£29.10	£23.65	£6.68^**^
Cost saving per respondent ^*^	NA	£5.45 (19%)	£22.42 (77%)^**^

^*^Compared to the paper survey (standard method employed in previous NMS)

^**^Cost per respondent and cost saving when including women living in England only; cost per respondent when including women living in all parts of the UK is £5.42 and the cost saving is £23.68 (81%)

The resulting costs per respondent were £29.10, £23.65 and £6.68 respectively (Table 4). Therefore, recruiting through ONS and including a paper questionnaire in all mailings was the most expensive survey and recruiting through social media and offering an online only questionnaire was the least expensive survey. Compared to the paper survey (the method used in all previous NMS), the push-to-web survey offered a cost saving of £5.45 (19%) per respondent (£19,971 overall) and the social media survey offered a cost saving of £22.42 (77%) per respondent (£62,380 overall).

## Discussion

In our 2020 NMS, an RCT of a paper survey and a push-to-web survey and a concurrent social media survey were conducted to compare different combinations of sampling and data collection methods with respect to response rate, respondent representativeness, external validity of prevalence estimates of selected maternity indicators, and financial costs.

The response rate was 3.5% higher in the standard paper survey compared to the push-to-web survey, in which women were encouraged to complete the questionnaire online. The difference was small but statistically significant and, with the current downward trend in response rates to surveys, even small improvements are important. The current findings, therefore, do not provide support for the use of push-to-web methods over standard paper methods based on response rates alone. The findings are in line with those of a recent US study which evaluated the effectiveness of push-to-web methods in a survey of new mothers and found that a traditional mailed survey yielded the highest response rate [12], yet contrary to previous studies which have provided evidence for push-to-web methods [9, 10]. In the current study, there were more responses in the paper and push-to-web surveys, both of which recruited through ONS, than in the social media survey, which recruited through online adverts over a period of three months.

Non-response bias in the surveys recruiting through ONS and self-selection bias in the social media survey meant that the respondents were not representative of all women giving birth in England during 2020 on key sociodemographic characteristics, such as age, ethnicity and level of deprivation. The women in the social media survey were even less representative of the target population on these sociodemographic characteristics compared to the women in the paper or push-to-web surveys. Therefore, these findings suggest that self-selection in surveys which recruit through social media may pose a greater threat to sample representativeness than high levels of non-response in surveys which recruit through defined sampling frames. This is consistent with systematic review findings that non-probability samples are less likely to be representative of the target population [4].

Weights were calculated and applied to the data in each survey to reduce the effect of non-response and self-selection bias, yet there was still bias in the survey estimates for some maternity indicators, when compared with population-based estimates available from national routine data. This may be due to the survey weights being based on limited sociodemographic variables, which do not fully explain the complex array of reasons for why women may have not responded to the survey [20]. There was no clear difference in the extent of bias across the paper, push-to-web and social media surveys, indicating that the reduced representativeness of the social media sample did not necessarily translate into more biased estimates, compared with the samples recruited through ONS.

The cost of recruiting through ONS was significantly higher than recruiting through social media. Compared to the paper survey, the standard method used in previous NMS, the social media survey offered a cost saving of more than twenty pounds (UK£) per respondent, which amounts to a substantial saving in large surveys. Consistent with previous findings [9], there was also a cost saving of approximately five pounds (UK£) in the push-to-web survey, compared to the standard method of sending paper questionnaires with each mailing. These cost savings could be redirected to enhance recruitment strategies, such as the use of prepaid incentives, which have been shown to be particularly effective in push-to-web surveys [21] or the offer of novel methods such as offline electronic questionnaire devices, which have recently been tested in the European Social Survey [22]. Investing in greater publicity and more targeted advertising for under-represented groups, which could in turn improve representativeness, may be an effective reallocation of funds in social media surveys. However, given that the paper survey resulted in more responses and a higher response rate (compared to the push-to-web survey), there is a trade-off which needs to be balanced in terms of maximising returns, enhancing other quality markers (such as representativeness) and minimising costs.

In addition to the potential cost saving, other advantages of social media surveys include the avoidance of coercion and intrusion, which can be inherent in survey invitations received via post. The self-selection sampling approach is potentially advantageous at a time when people may be feeling increasingly bombarded with requests for personal information. Related to this is the feasibility of conducting social media surveys without the requirement for any personal information, which avoids the potential annoyance caused when invitation recipients are unclear about how their details have been accessed. An additional advantage of surveys carried out solely online is the lower environmental impact, which is an increasingly recognised outcome.

There are also disadvantages of social media surveys. Although there are examples of surveys which have recruited very large samples through social media, such as a recent Spanish study on the impact of Covid-19 on the entire adult population, which achieved almost 142,000 responses within one week [23], most social media surveys employ narrower eligibility criteria meaning the pool of potential participants is substantially reduced. Maternity surveys which have recruited through social media have typically achieved samples ranging from several hundred women [24, 25] to between 1,000 and 4,000 women (most less than 1,500) [26–29], even with relatively broad eligibility criteria (e.g. pregnant women, women who are breastfeeding, women with infants aged 0–36 months). Our social media survey recruited 1,622 women overall, which is a relatively sizeable sample, especially considering the narrower eligibility criteria (women who had given birth in the UK during a six-month period). Although broader criteria may increase the pool of potential participants, data quality may be impacted and the scope to address particular topics may be limited. For example, accuracy of recall of maternity experiences may diminish as time since pregnancy and childbirth increases. Similarly, retrospective evaluation of maternal outcomes such as postnatal mental health and breastfeeding practices may become more challenging and less valid beyond the postnatal period.

Broader eligibility criteria may also increase the likelihood of obtaining heterogeneous samples. For example, in the current study the women recruited through social media gave birth over a six-month period whereas the women recruited though ONS gave birth during a two-week period. In a survey exploring maternity experiences during a fast evolving event like the Covid-19 pandemic, timing is critical. The rapid changes in guidelines and practice as more information became available are likely to have had an impact on women’s care [30]. Hence, women giving birth at different times during the pandemic, even several weeks apart, may have reported differing experiences. Therefore, social media surveys may be effective for recruiting from very large target populations, particularly when the topic is of wide interest, yet less effective for recruiting targeted samples for surveys on specific topics or at particular points in time. For targeted samples, a longer recruitment window may be needed to recruit sufficient numbers of participants, which might then negate some of the aforementioned cost benefits. An additional disadvantage of social media surveys, as opposed to surveys with defined sampling frames, is verifying that respondents meet the eligibility criteria when relying solely on self-screening for entry to the survey.

On balance, despite some clear advantages of social media surveys, there are also numerous limitations that should be taken into account when considering social media surveys as an alternative to traditional survey methods. The current findings have implications for other UK and international organisations who conduct large population-based surveys, and who may be considering reviewing traditional survey methods in light of the availability of more contemporary and seemingly cost-effective methods.

The main strength of this analysis is that the three surveys were carried out concurrently with many consistent methodological components, which enables the sampling and data collection methods to be reliably compared. The inclusion of the RCT, which used ONS birth registration data to compare the standard paper and push-to-web surveys, is a particular strength and the large sample size in the RCT allows precise estimates to be calculated and the results to be generalised to other similar surveys. Furthermore, all surveys were sufficiently large to allow the representativeness of the respondents to be assessed. The main limitation of the analysis is that the variables used to construct the survey weights did not explain all of the non-response and self-selection bias [3]. Successfully reducing bias in samples with low response rates and in self-selecting samples requires adjusting for the correct auxiliary variables [31]. These are likely to include more than core sociodemographic characteristics; careful consideration of the factors that differentiate the respondents from the target population and their association with the outcomes of interest is key [20].

## Conclusions

The standard paper survey generated the most returns and the highest response rate. Push-to-web surveys may offer a cost saving compared to paper surveys, without compromising the representativeness of respondents or external validity of survey estimates, yet they do not necessarily result in higher response rates. Social media surveys offer a significant cost saving compared to paper and push-to-web surveys, but the respondents may be less likely to represent the target population and it may not always be possible to recruit sufficient numbers. However, reduced representativeness does not necessarily introduce more bias in survey estimates, particularly when survey data are weighted to account for non-response or self-selection bias. Future research should explore whether targeted recruitment to increase the inclusion of under-represented groups could increase representativeness and validity in social media surveys, thus demonstrating the viability of using social media surveys to collect nationally representative maternity survey data.

## Data Availability

The datasets used and/or analysed during the current study are available from the corresponding author on reasonable request.

## Abbreviations

CI: Confidence interval
IMD: Index of Multiple Deprivation
NMS: National Maternity Survey
NPEU: National Perinatal Epidemiology Unit
ONS: Office for National Statistics
