# Correction: Impact of sampling and data collection methods on maternity survey response: a randomised controlled trial of paper and push-to-web surveys and a concurrent social media survey

**DOI:** 10.1186/s12874-023-01852-5

**Published:** 2023-01-28

**Authors:** Siân Harrison, Fiona Alderdice, Maria A. Quigley

**Affiliations:** NIHR Policy Research Unit in Maternal and Neonatal Health and Care, National Perinatal Epidemiology Unit, Nuffield Department of Population Health, University of Oxford, Old Road Campus Headington, Oxford, OX3 7LF UK

**Correction: BMC Medical Research Methodology 23, 10 (2023)**

**https://doi.org/10.1186/s12874-023-01833-8**

Following publication of the original article [1], the author requested to correct the word "primiparous" to "multiparous" in the Abstract results and under the paragraph “Representativeness of respondents” in the main results section.

The original article has been updated.
