# Correction: Impact of sampling and data collection methods on maternity survey response: a randomised controlled trial of paper and push‑to‑web surveys and a concurrent social media survey

**DOI:** 10.1186/s12874-024-02216-3

**Published:** 2024-04-29

**Authors:** Siân Harrison, Fiona Alderdice, Maria A. Quigley

**Affiliations:** NIHR Policy Research Unit in Maternal and Neonatal Health and Care, National Perinatal Epidemiology Unit, Nuffield Department of Population Health, University of Oxford, Old Road Campus Headington, Oxford, OX3 7LF UK

**Correction: BMC Med Res Methodol 23, 10 (2023)**

**https://doi.org/10.1186/s12874-023-01833-8**

Following publication of the original article [1], the authors reported an error in the Fig. 4: the colours in the pie charts in Fig. 4 do not all correspond with the legend. See the Fig. 4 corrected.

**Fig. 4:**
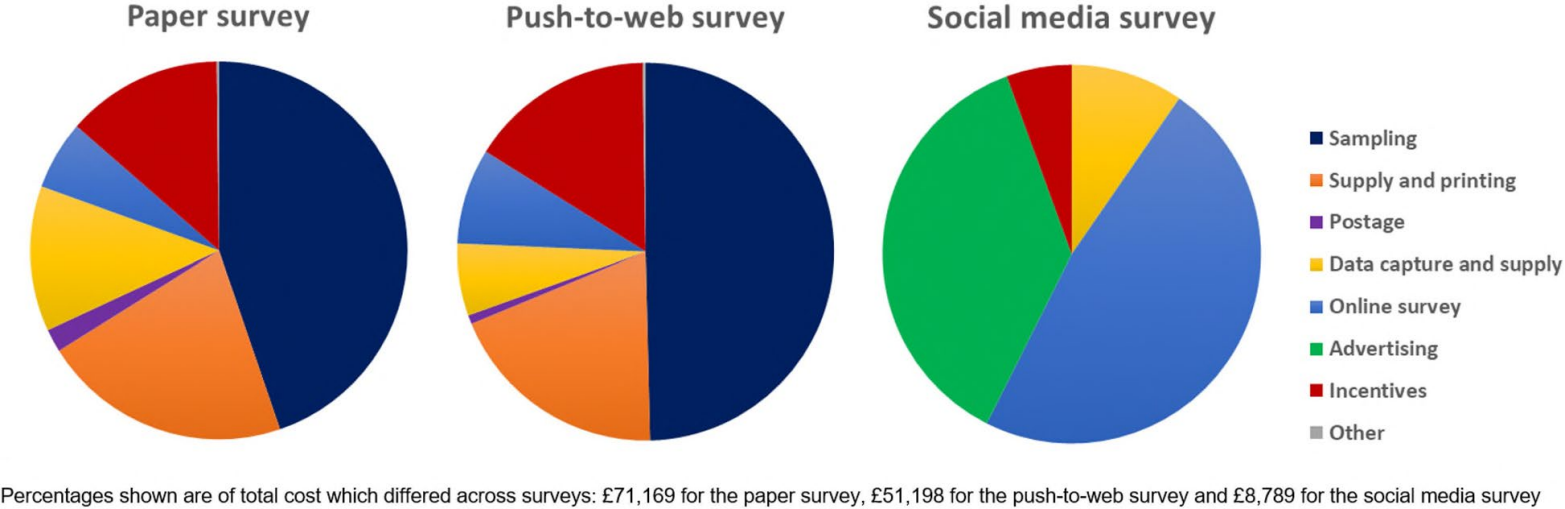
Breakdown of total costs across the surveys

The original article [1] has been updated.
